# Optimizing Broiler Performance and Feed Cost Efficiency: Impact of 1,3-Diacylglycerol Supplementation at Different Energy Levels

**DOI:** 10.3390/vetsci12070633

**Published:** 2025-07-02

**Authors:** Wei Han Zhao, Se Yeon Jang, In Ho Kim

**Affiliations:** 1 Department of Animal Biotechnology, Dankook University, Cheonan 31187, Republic of Korea; zhaoweihan57@gmail.com (W.H.Z.); tpdus9860@naver.com (S.Y.J.); 2 Smart Animal Bio Institute, Dankook University, Cheonan 31187, Republic of Korea

**Keywords:** 1,3-diacylglycerol, broiler, energy, growth performance, meat quality

## Abstract

We evaluated the effects of supplementing 1,3-diacylglycerol (1,3-DAG) in diets with different energy levels on the growth performance, nutrient digestibility, excreta scores, rectal temperature, meat quality, and blood parameters of broilers. A total of 576 one-day-old Ross 308 broilers (initial BW: 47.65 ± 0.51 g) were used in a 35-day feeding trial. The broilers were randomly assigned to four treatment groups (144 birds per group), with eight cages per group and 18 birds per cage, consisting of 9 males and 9 females. A 2 × 2 factorial design was employed, with two dietary energy levels (normal and reduced by 100 kcal/kg) with or without 0.075% 1,3-DAG supplementation. The results showed that compared with the diets without 1,3-DAG, the broilers receiving 1,3-DAG supplementation exhibited significantly greater body weight gain (BWG) and overall body weights (BWs) from days 10 to 35, along with a lower feed conversion ratio (FCR) (*p* < 0.05). In contrast, the low-energy diets without 1,3-DAG supplementation resulted in reduced growth performance, an increased FCR, higher drip loss, and lower total cholesterol levels. Notably, the rectal temperature and excreta scores were not affected by dietary energy levels or 1,3-DAG supplementation. In conclusion, while low-energy diets negatively impact growth and meat quality, 1,3-DAG supplementation enhances energy digestibility and growth performance, partially alleviating the adverse effects of reduced-energy diets and potentially lowering feed costs without compromising growth.

## Simple Summary

Feed energy is a major cost in broiler production. Reducing dietary energy levels can lower feed costs but often leads to poor growth performance. This study explored whether supplementing 1,3-diacylglycerol, a lipid with emulsifying properties, could improve the performance of broilers fed low-energy diets. The results showed that adding 1,3-diacylglycerol to the diet improved growth, feed efficiency, and nutrient digestibility, even under reduced-energy conditions. These findings suggest that 1,3-diacylglycerol is a promising feed additive that can help reduce feed costs without compromising broiler performance or meat quality.

## 1. Introduction

Energy is a major cost factor in broiler feed. Additionally, the potential to lower feed costs by formulating low-energy diets without compromising broiler growth has become a topic of considerable interest [1]. Lipids, as a key energy source in poultry feed, not only enhance the energy density of the diet but also improve feed palatability and growth performance and reduce feed dust [2]. Fats exert various metabolic effects in poultry, and thus dietary energy levels are often modified by adding fats. This adjustment can create potential interactions that influence the metabolizable energy (ME) content of the diet, enhance the absorption and utilization of other nutrients, and ultimately lead to beneficial outcomes [3]. In the early stages of chick development, the immature digestive system limits the digestion and absorption of fats. Specifically, the restricted production and secretion of bile acids and lipase further reduce the efficient utilization of lipids. Unabsorbed fats are often excreted, leading to energy waste [4]. Studies have shown that the inclusion of exogenous emulsifiers can enhance lipid absorption, increase fat digestion efficiency, and improve overall energy utilization. These effects are particularly notable in low-energy diets and fats with lower digestibility [5]. Diacylglycerol (DAG) is a unique lipid structure widely recognized for its emulsifying properties in the food and pharmaceutical industries. In particular, 1,3-diacylglycerol (1,3-DAG) has been shown to enhance fat metabolism and lower serum lipid levels [6]. Additionally, research has shown that DAG rich in medium-chain fatty acids can be rapidly absorbed and metabolized for energy, significantly reducing fat accumulation in the body, and it also enhances energy utilization efficiency, growth performance, and nutrient digestibility [7]. However, the specific mechanisms of DAG in poultry production, particularly its effects when combined with low-energy diets, require further investigation. This study aims to explore the potential application of 1,3-DAG in diets with varying energy levels, systematically evaluating its effects on broiler growth performance, nutrient digestibility, the rectal temperature, excreta scores, blood parameters, and meat quality. The research hypothesis is that the addition of 1,3-DAG can enhance broiler growth performance and meat quality by improving fat metabolism, promoting nutrient absorption, and increasing energy utilization efficiency. The findings of this research will not only enhance the understanding of the practical value of 1,3-DAG in poultry production but also provide a theoretical foundation for developing efficient and cost-effective feed formulations.

## 2. Materials and Methods

Experimental protocols describing the management and care of animals were reviewed and approved by the Animal Care and Use Committee of Dankook University (Approval No. DK-1-2426. Approval Date: 14 June 2024) in the Republic of Korea.

### 2.1. Experimental Material

The 1,3-diacylglycerol (1,3-DAG) used in this experiment was provided by a commercial company (K-MAX, Il Shin Wells, Seoul, Republic of Korea) and contained 55% 1,3-DAG. It was composed of 70% medium-chain triglycerides (MCTs) and 30% long-chain triglycerides (LCTs). The fatty acid profile consisted of lauric acid, capric acid, and a combination of myristic and palmitic acids at a ratio of 50:20:30, respectively. At an inclusion level of 0.075%, 1,3-DAG was estimated to provide approximately 2.88 kcal/kg of metabolizable energy.

### 2.2. Experimental Design, Animals, and Diets

**Figure 1:**
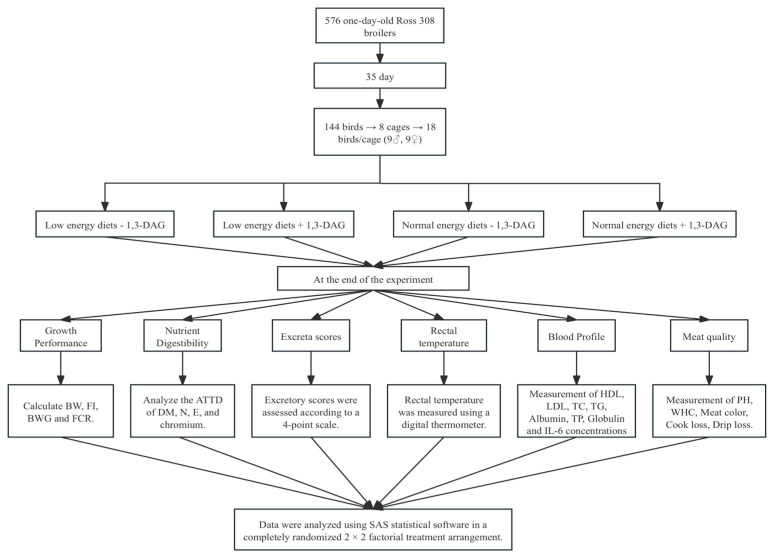
Experimental flow design diagram.

A total of 576 broiler chickens (one day old, Ross 308) were obtained from a commercial hatchery. Broilers with similar body weights (47.65 ± 0.51 g) were randomly allocated into four groups (144 birds per group, with 8 cages per group and 18 birds per cage, consisting of 9 males and 9 females per cage) The experimental design is shown in Figure 1. The broiler chickens were housed in a temperature-controlled room with three-tier stainless steel battery cages (124 cm wide × 64 cm long × 40 cm high). The experimental animal housing was equipped with a single-sided feeder (35 cm in diameter) and nipple drinkers (five nipples per cage) to ensure ad libitum access to feed and water throughout the experimental period. The lighting schedule was set to 22 h of light and 2 h of darkness for the entire 35-day period. The ambient temperature was maintained at 33 °C during the first week and then gradually reduced to 20 °C by the fifth week. The relative humidity was gradually increased from 60% (d 1–21) to 70% (d 22–35). The facility was inspected four times daily (8:00 a.m., 12:00 p.m., 4:00 p.m., and 8:00 p.m.) to monitor the feed availability, water supply, and health status. The broiler chickens were fed a corn- and soybean-based basal diet for 35 days, which was divided into three phases: Phase 1 (d 1–9), Phase 2 (d 10–21), and Phase 3 (d 21–35) (Table 1). The experimental diets were provided in mashed form, with feed and additives mixed using a feed mixer (Daedong Tech, DDK-801, Anyang, South Korea). The experimental diets were prepared according to the nutritional recommendations for Ross 308 [8]. A 2 × 2 factorial treatment arrangement was used, consisting of two energy levels (normal energy diet and reduced energy diet) and the inclusion or exclusion of 0.075% 1,3-DAG.

### 2.3. Growth Performance

The body weight (BW) and feed intake (FI) per cage were recorded on days 9, 20, and 35. The feed conversion ratio (FCR) was calculated as the feed intake divided by the body weight gain (BWG). Mortality was recorded daily, and the mortality percentage was calculated for the entire experimental period.

### 2.4. Nutrient Digestibility

Prior to excreta collection, all broilers in eight cages per treatment group were fed diets containing 0.2% chromium oxide (Cr_2_O_3_) for seven consecutive days (from day 28 to day 34). The diets were mixed well using a feed mixer (Daedong Tech, DDK-801, Anyang, Republic of Korea) to determine nutrient utilization. On day 35, excreta samples were collected 4–8 h after feeding. All excreta samples were pooled by cage and thoroughly mixed, and representative samples were stored at −20 °C. The samples were dried in a drying oven (Daihan Scientific Co., Ltd., WOF-L800, Seoul, Republic of Korea) at 70 °C for 72 h and then ground to pass through a 1-mm sieve. The dry matter (DM), nitrogen (N), and energy (E) contents of the feed and excreta samples were analyzed using AOAC (2010) methods [9]. Chromium concentrations were determined via ultraviolet spectrophotometry, and the energy content was measured by determining the heat of combustion using a bomb calorimeter (Parr 6100, Parr Instrument Co., Moline, IL, USA). The following formula was used to calculate the apparent total tract digestibility (ATTD):

$$ATTD(\%)=[1-\{(N_{f}\times C_{d})/(N_{d}\times C_{f}\}]\times 100$$

where *N_f_* indicates the concentration in the feces (% DM), *N_d_* indicates the nutrient concentration in the diets (% DM), *C_f_* indicates the chromium concentration in the feces (% DM), and *C_d_* indicates the chromium concentration in the diets (% DM).

### 2.5. Excreta Scores

**Figure 2:**
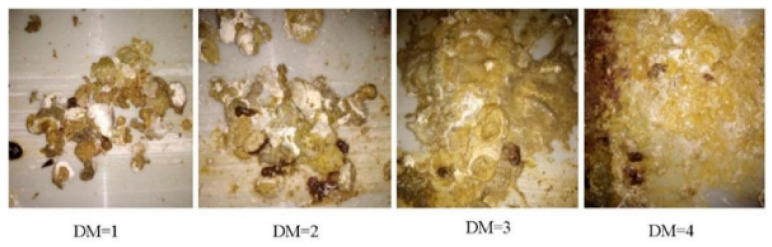
Excreta scores.

On day 35, excreta scoring was conducted as illustrated in Figure 2. The excreta scores for each cage ranged from 1 to 4 (where 1 means the dropping is spherical, with a large cluster in the middle and a small amount of granular distribution around it and almost no water in the excrement; 2 means excreta irregularity, containing a small amount of undigested feed and water and most of the middle of the excreta mass; 3 means the feed is more undigested, as there is a small granular distribution amount and water content and the excreta are formless; and 4 means the feces are completely unformed, containing undigested feed particles and being spread almost like water on the feces scraping board) [10]. Old feces were first removed from the tray, and clean white paper was placed on it. Two independent evaluators then performed visual scoring based on the aforementioned criteria, and the average score was recorded.

### 2.6. Rectal Temperature

At the end of the experiment, the rectal temperature was measured. Eight chickens were randomly selected from each treatment group (four females and four males, with one bird per replicate cage), and their rectal temperatures were measured using a digital thermometer with an accuracy of ±0.1 °C (Thermalert TH-5, Physitemp Instruments Inc., Clifton, NJ, USA). The thermistor probe was inserted into the chick’s rectum to a depth of 2 cm from the anus. It took approximately 5 s to measure the rectal temperature after inserting the probe.

### 2.7. Blood Profile

At the end of the experiment, 8 chickens were randomly selected from each treatment group (4 females and 4 males, with 1 bird per cage and 32 birds in total), and approximately 5 mL of blood was collected from the wing vein using a sterile syringe. The blood samples were then transferred to vacuum tubes (Becton Dickinson Vacutainer Systems, Franklin Lakes, NJ, USA) to obtain serum and whole blood samples. The serum samples were centrifuged at 3000× *g* for 15 min, after which the serum was removed and stored at −20 °C until analysis. The serum’s total cholesterol, high-density lipoprotein (HDL), low-density lipoprotein (LDL), and triglyceride levels were measured using an automatic blood analyzer (ADVIA 120, Bayer, Tarrytown, NY, USA) and commercial reagent kits (Sigma Diagnostics, St. Louis, MO, USA: MAK043, MAK045, TR0100). The total protein, albumin, globulin, and IL-6 were analyzed using an automatic biochemical analyzer (HITACHI 747, Hitachi, Tokyo, Japan).

### 2.8. Meat Quality

On day 35, all broilers in each treatment group were fasted for 8 h before blood collection. Sixteen broilers per group (1 female and 1 male per cage, with 64 birds in total) were randomly selected for meat quality assessment. After blood sample collection, the birds were euthanized via cervical dislocation. The Hunter CIE lightness (L*), redness (a*), and yellowness (b*) of the breast muscle were measured using a Minolta CR410 colorimeter (Konica Minolta Sensing, Inc., Tokyo, Japan). These values were calculated as the average of three positions on the surface of each sample. The pH of the breast muscle was measured using a pH meter (Fisher Scientific, Waltham, MA, USA), with two measurements taken for each sample. The water-holding capacity (WHC), cooking loss, and drip loss were evaluated according to the method of Lee et al. (2022) [11].

### 2.9. Statistical Analysis

The data were analyzed using SAS 9.4 statistical software (SAS Inst. Inc., Cary, NC, USA, 2002) with a completely randomized 2 × 2 factorial treatment arrangement, where the cage served as an experimental unit. The main effects of 1,3-DAG and the energy content, as well as their interaction, were tested. The significance level was set at *p* < 0.05, and 0.05 < *p* < 0.10 was considered a trend.

## 3. Results

The effects of dietary treatments on growth performance are shown in Table 2. Compared with the treatment group without 1,3-DAG supplementation, the group supplemented with 1,3-DAG showed significantly higher BWG during days 10–20, days 21–35, and the overall experimental period (*p* < 0.05). Additionally, the BW was significantly higher during the overall period (*p* < 0.05), and the FCR was significantly lower during days 10–20, days 21–35, and the overall period (*p* < 0.05). The FCR tended to decrease during days 1–9 (*p* < 0.10). However, compared with the broilers fed a normal-energy diet, the broilers fed a low-energy diet showed significantly lower BWG during the overall period (*p* < 0.05), with a tendency for reduced BWG during days 10–20 and days 21–35. Body weights were significantly lower during the entire feeding period (*p* < 0.05), and the FCR was significantly higher during days 1–9, days 10–20, days 21–35, and the entire experimental period (*p* < 0.05). There was no significant interaction between the dietary energy levels and 1,3-DAG supplementation regarding the growth performance of the broilers (*p* > 0.05).

The effects of dietary treatments on nutrient digestibility are shown in Table 3. Compared with the low-energy diet without 1,3-DAG supplementation, the normal-energy diet supplemented with 1,3-DAG significantly improved energy digestibility (*p* < 0.05). There was no significant interaction between dietary energy levels and 1,3-DAG supplementation regarding the nutrient digestibility of the broilers (*p* > 0.05).

The effects of the dietary treatments on the excreta scores are summarized in Table 4. In this study, the dietary energy levels and 1,3-DAG supplementation had no significant effects on or interactions with the excreta scores for the broilers (*p* > 0.05).

The effects of dietary treatments on the rectal temperature are shown in Table 5. In this study, the dietary energy levels and 1,3-DAG supplementation had no significant effects on or interactions with the rectal temperatures of the broilers (*p* > 0.05).

The effects of the dietary treatments on meat quality are shown in Table 6. On day 7 post-slaughter, drip loss was significantly increased in the broilers fed a low-energy diet without 1,3-DAG compared with those fed a normal-energy diet supplemented with 1,3-DAG (*p* < 0.05). There was no significant interaction between the dietary energy levels and 1,3-DAG supplementation regarding the meat quality traits of the broilers.

The effects of dietary treatments on the blood parameters are shown in Table 7. Compared with the broilers fed a normal-energy diet without 1,3-DAG supplementation, those fed a low-energy diet supplemented with 1,3-DAG had significantly lower total cholesterol levels (*p* < 0.05). There was no significant interaction between the dietary energy levels and 1,3-DAG supplementation regarding the blood parameters of the broilers.

## 4. Discussion

The development of modern broiler genetic lines has enhanced their growth potential and increased their demand for high energy intake. To meet this requirement, high-energy feed is essential, with fats and oils commonly incorporated into broiler diets to enhance energy density. This is because fats provide at least twice the energy of carbohydrates and proteins [12]. Previous studies have demonstrated that the apparent ME and total fat metabolizable in broilers are influenced by age, particularly during the first week after hatching, but they typically improve after two weeks [13]. Consequently, the inclusion of exogenous emulsifiers in diets has emerged as a potential supplement to enhance fat utilization and maintain the high productivity of broilers [14]. Emulsifiers are especially important in commercial broiler production under energy-limited dietary conditions [15]. Previous studies have shown that adding emulsifiers can partially compensate for the negative effects of low-energy diets on growth performance, bringing broiler growth close to that of the normal-energy diet group and also reducing feeding costs [16]. Studies have shown that incorporating 1,3-DAG emulsifiers at concentrations of 0.075%, 0.10%, and 0.15% in low-energy diets positively affects body weight gain, feed intake, and the feed conversion ratio in broilers [7]. Our findings are consistent with these results, as the supplementation of 1,3-DAG led to a significant improvement in growth performance. However, some reports suggest that adding 0.1% DAG emulsifiers to the diet had no significant impact on the growth performance of pigs [17]. This discrepancy may be related to differences in animal species or the specific type of DAG emulsifier used. The isomers of DAG mainly include 1,2-DAG and 1,3-DAG, and structural differences between these isomers may affect their emulsifying properties and fat metabolism efficiency. Additionally, these two isomers differ in their synthesis and metabolic pathways, as 1,2-DAG is primarily generated during the breakdown of dietary triacylglycerols and can be incorporated into cellular membranes, affecting membrane fluidity and signaling pathways. On the other hand, 1,3-DAG is often produced during the synthesis of specific lipids and may be preferentially utilized in energy production or fat storage, influencing lipid metabolism differently [18]. Studies have also shown that supplementing low-energy diets with 1,3-DAG can significantly improve energy digestibility by reducing feed energy wastage through the rapid metabolism of medium-chain fatty acids (MCFAs) [7]. This is consistent with our study, where supplementation with 1,3-DAG significantly increased the digestibility of energy. The improvement in energy may be attributed to the MCFA present in DAG, which are more readily metabolized for energy. However, studies have indicated that broilers fed a diet with a mixture of emulsifiers showed no differences in dry matter, nitrogen, or energy digestibility [4]. Other products or differences in the source of 1,3-DAG occur. In poultry farming, dietary energy levels can be adjusted to regulate growth performance and feed costs [19]. Lowering dietary energy levels has been shown to reduce growth performance in broilers, although no significant difference in FI is typically observed [20,21], which is consistent with our findings. In our study, although the energy difference between the normal- and low-energy diets was only 100 kcal/kg, the broilers fed the low-energy diet exhibited significantly lower BWG throughout the experimental period compared with those on the normal-energy diet. Additionally, the FCR was significantly higher during days 1–9, days 10–20, days 21–35, and the overall experimental period. The energy provided by the diet is primarily used for maintenance and production. When broilers are fed a low-energy diet, the energy is mainly allocated for maintenance, which can impair growth. This may help explain the decrease in BWG and the increase in FCR observed in this study [22]. Dietary energy concentration also influences broiler FI. Broilers can adjust their feed intake to regulate energy consumption in response to changes in diet energy concentration [23,24]. Therefore, broilers fed a reduced-energy diet tend to consume more feed to compensate for the energy deficit. However, in this study, there was no significant difference in FI based on energy levels, which may be due to environmental factors, such as the temperature, humidity, and stocking density, influencing broiler feeding behavior. Under consistent rearing conditions, broilers may not adjust their feed intake in response to changes in energy levels [25]. In this study, dietary supplementation with 1,3-DAG had no significant effect on the rectal temperatures or fecal scores in the broiler chickens. This result may be attributed to the primary role of 1,3-DAG in enhancing fat digestion, absorption, and energy utilization rather than directly influencing thermoregulation or gut health-related physiological mechanisms. Although fat metabolism can impact the body’s heat balance, the relatively low supplementation level of 1,3-DAG in this study may not have been sufficient to induce significant changes in rectal temperature [26]. Additionally, fecal scores are mainly influenced by dietary composition, gut microbiota, and water metabolism. Since 1,3-DAG primarily functions in fat emulsification and absorption, it may not have significantly altered the physical characteristics of the excreta [27]. Future research should explore whether different levels of 1,3-DAG supplementation under higher ambient temperatures or varying dietary formulations could have potential effects on the rectal temperature and fecal quality.

Studies have reported that compared with broilers fed a normal-energy diet, those on a low-energy diet may experience increased protein catabolism due to energy deficiency. This can lead to the degradation of muscle cell structures, reducing the water-binding capacity of muscle tissue and consequently increasing drip loss in broilers [28]. Our findings align with this, as feeding a low-energy diet significantly increased drip loss. Additionally, although reduced-energy diets led to lower final body weights in the broilers, they did not affect carcass composition when compared with the control diet [29,30]. This contrasts with our study, where changes in the ratio of other nutrients (such as protein and fat) in the low-energy diet may explain the observed differences. Studies have also shown that high-energy diets typically contain more fats and oils, which enhance the intestinal absorption of dietary lipids and increase the availability of cholesterol precursors [31]. This, in turn, promotes hepatic cholesterol synthesis, elevating the total cholesterol, HDL cholesterol, and LDL cholesterol levels in broiler chickens. Additionally, low cholesterol may impair immune response, thereby increasing the risk of disease infection in broilers [32]. This is consistent with our findings, where a low-energy diet significantly reduced the total cholesterol levels in the broilers, likely due to alterations in lipid metabolism resulting from reduced dietary fat and energy intake. When energy intake is restricted, stored lipids may be utilized more efficiently, leading to lower circulating cholesterol levels [33]. However, some studies have reported that the dietary energy content does not affect the HDL, LDL, total cholesterol, and triglyceride concentrations in broilers [23,34]. This is inconsistent with our study results, which may be attributed to the total cholesterol measurement technique, a method that is difficult to standardize and characterized by variability and high coefficients of variation, which reduces the consistency of the results. In the poultry industry, low-energy diets can help reduce production costs by modifying the energy composition and supplementing emulsifiers. In our study, supplementation with 1,3-DAG and varying dietary energy levels showed no interactive effects on the growth performance, nutrient digestibility, excreta scores, rectal temperature, meat quality, or blood lipid indicators in broiler chickens. This is consistent with previous studies that also found no interaction between emulsifiers and dietary energy levels regarding growth performance, nutrient digestibility, blood lipid indicators, and meat quality [4]. However, some studies have reported that emulsifiers interact with dietary energy levels to influence broiler growth performance [35]. This difference may be attributed to the chemical composition of the emulsifier and its distinct mechanisms of action in fat emulsification and absorption. Moreover, lower supplementation levels of emulsifiers may not be sufficient to significantly improve lipid digestibility, which could explain the absence of notable interaction.

## 5. Conclusions

The inclusion of 1,3-DAG in a diet can improve the growth performance and nutrient digestibility of broiler chickens. However, reducing the energy by 100 kcal/kg in the diet decreases growth performance and total cholesterol levels and increases drip loss in meat. The use of 1,3-DAG can offset the negative effects of reduced energy, thereby lowering production costs. Further research is needed to explore the interaction between 1,3-DAG and energy levels in broiler chicken diets.

## Figures and Tables

**Table 1:** Ingredient composition of experimental diets on as-fed basis.

Item	(Starter) ^1^		(Grower) ^1^		(Finisher) ^1^	
	PC	NC	PC	NC	PC	NC
Ingredients (%)						
Corn	57.34	60.62	62.68	66.00	66.80	69.96
Soybean meal	24.32	23.73	20.30	19.69	18.97	18.40
Corn gluten meal	10.00	10.00	10.00	10.00	8.00	8.00
Tallow	4.12	1.42	3.02	0.29	2.58	0.01
MDCP	1.88	1.88	1.94	1.94	1.68	1.65
Limestone	1.49	1.50	1.24	1.24	1.15	1.16
Salt	0.20	0.20	0.20	0.20	0.20	0.20
Methionine (99%)	0.13	0.13	0.10	0.10	0.09	0.09
Lysine (78%)	0.29	0.29	0.29	0.31	0.30	0.30
Mineral mix ^2^	0.10	0.10	0.10	0.10	0.10	0.10
Vitamin mix ^3^	0.10	0.10	0.10	0.10	0.10	0.10
Choline (25%)	0.03	0.03	0.03	0.03	0.03	0.03
Total	100.00	100.00	100.00	100.00	100.00	100.00
Calculated value						
Crude protein, %	23.00	23.00	21.50	21.50	20.00	20.00
Ca, %	1.10	1.10	1.00	1.00	0.90	0.90
P, %	0.70	0.70	0.70	0.70	0.64	0.64
Available P, %	0.45	0.45	0.45	0.45	0.40	0.40
Lys, %	1.20	1.20	1.10	1.10	1.05	1.05
Met, %	0.54	0.54	0.50	0.50	0.46	0.46
ME, kcal/kg	3200	3100	3200	3100	3200	3100
FAT, %	6.72	4.19	5.80	3.24	5.46	3.05
Fiber, %	2.34	2.39	2.30	2.35	2.31	2.36
Ash, %	6.41	6.42	6.03	6.03	5.54	5.52

^1^ Starter phase = d 0–9; grower phase = d 9–20; and finisher phase = d 21–35. Abbreviations: PC = positive control (normal-energy diet); NC = negative control (normal-energy diet of 100 kcal/kg); MDCP = mono dicalcium phosphate; ME = metabolizable energy; LYS = lysine; MET = methionine. ^2^ Provided per kg of complete diet: 37.5 mg Zn (as ZnSO_4_); 37.5 mg Mn (as MnO_2_); 37.5 mg Fe (as FeSO_4_·7H_2_O); 3.75 mg Cu (as CuSO_4_·5H_2_O); 0.83 mg I (as KI); and 0.23 mg Se (as Na_2_SeO_3_·5H_2_O). ^3^ Provided per kg of complete diet: 15,000 IU of vitamin A; 3750 IU of vitamin D_3_; 37.5 IU of vitamin E; 2.55 mg of vitamin K_3_; 3 mg of thiamin; 7.5 mg of riboflavin; 4.5 mg of vitamin B_6_; 24 μg of vitamin B_12_; 51 mg of niacin; 1.5 mg of folic acid; 0.2 mg of biotin; and 13.5 mg of Ca-pantothenate.

**Table 2:** Effect of 1,3-diacylglycerol supplementation in diets with different energy levels on the growth performance of broilers ^1^.

Energy	Low Energy		Normal Energy		SEM	*p* Value		
1,3-DAG	−	+	−	+				
Items						Energy	1,3-DAG	Interaction
d 1–9								
BWG, g	199	202	202	206	4	0.381	0.456	0.948
FI, g	217	216	215	215	5	0.728	0.895	0.876
FCR	1.093 ^a^	1.067 ^ab^	1.06 ^ab^	1.04 ^b^	0.01	0.027	0.074	0.797
d 10–20								
BWG, g	713 ^b^	740 ^ab^	734 ^ab^	761 ^a^	11	0.080	0.022	0.987
FI, g	939	925	916	914	17	0.332	0.645	0.755
FCR	1.315 ^a^	1.251 ^ab^	1.251 ^ab^	1.203 ^b^	0.02	0.021	0.019	0.729
d 21–35								
BWG, g	993 ^b^	1057 ^ab^	1048 ^ab^	1089 ^a^	22	0.064	0.026	0.622
FI, g	1857	1811	1803	1789	36	0.307	0.415	0.673
FCR	1.869 ^a^	1.718 ^b^	1.724 ^b^	1.644 ^b^	0.03	0.002	0.001	0.274
Overall								
BW, g	1953 ^b^	2047 ^ab^	2031 ^ab^	2104 ^a^	28	0.027	0.008	0.721
BWG, g	1906 ^b^	2000 ^ab^	1984 ^ab^	2056 ^a^	28	0.027	0.008	0.715
FI, g	3013	2952	2934	2917	51	0.279	0.456	0.676
FCR	1.581 ^a^	1.477 ^b^	1.479 ^b^	1.420 ^b^	0.02	<0.0001	<0.0001	0.176
Mortality	3.47	4.17	4.17	3.47	-			

^1^ Abbreviations: (Each mean represents values from 8 replicates (18 birds/replicate)). 1,3-DAG = 1,3-diacylglycerol; BWG = body weight gain; FI = feed intake; FCR = feed conversion ratio; BW = body weight; SEM = standard error of means. ^a,b^ Means in the same row with different superscripts differ significantly (*p* < 0.05).

**Table 3:** Effect of 1,3-diacylglycerol supplementation in diets with different energy levels on nutrient digestibility of broilers ^1^.

Energy	Low Energy		Normal Energy		SEM	*p* Value		
1,3-DAG	−	+	−	+				
Items, %						Energy	1,3-DAG	Interaction
Finish								
Dry matter	72.01	71.73	71.35	72.87	0.95	0.811	0.530	0.368
Nitrogen	69.20	69.81	69.72	70.76	0.55	0.208	0.161	0.708
Energy	69.72 ^b^	70.91 ^ab^	70.55 ^ab^	72.33 ^a^	0.65	0.110	0.043	0.662

^1^ Abbreviations: (Each mean represents values from 8 replicates (18 birds/replicate)). 1,3-DAG = 1,3-diacylglycerol; SEM = standard error of means. ^a,b^ Means in the same row with different superscripts differ significantly (*p* < 0.05).

**Table 4:** Effect of 1,3-diacylglycerol supplementation in diets with different energy levels on excreta scores of broilers ^1^.

Energy	Low Energy		Normal Energy		SEM	*p* Value		
1,3-DAG	−	+	−	+				
Items						Energy	1,3-DAG	Interaction
Excreta score								
Finish	1.56	1.50	1.50	1.44	0.19	0.750	0.750	1.000

^1^ Abbreviation: (Each mean represents values from 8 replicates (18 birds/replicate)). 1,3-DAG = 1,3-diacylglycerol; SEM = standard error of means.

**Table 5:** Effect of 1,3-diacylglycerol supplementation in diets with different energy levels on rectal temperatures of broilers ^1^.

Energy	Low Energy		Normal Energy		SEM	*p* Value		
1,3-DAG	−	+	−	+				
Items, °C						Energy	1,3-DAG	Interaction
Rectal temperature								
Finish	41.86	41.95	42.00	42.03	0.12	0.397	0.652	0.802

^1^ Abbreviations: (Each mean represents values from 8 replicates (18 birds/replicate)). 1,3-DAG = 1,3-diacylglycerol; SEM = standard error of means.

**Table 6:** Effect of 1,3-diacylglycerol supplementation in diets with different energy levels on meat quality of broilers ^1^.

Energy	Low Energy		Normal Energy		SEM	*p* Value		
1,3-DAG	−	+	−	+				
Items						Energy	1,3-DAG	Interaction
Finish								
pH	5.73	5.64	5.86	5.80	0.11	0.247	0.554	0.886
WHC, %	48.24	50.28	50.99	51.42	1.75	0.290	0.497	0.657
Meat color								
L*	55.23	51.57	52.19	50.03	3.69	0.547	0.446	0.842
a*	5.20	5.19	5.25	5.33	0.72	0.893	0.962	0.949
b*	14.34	15.06	15.11	13.20	1.40	0.705	0.680	0.367
Cook loss, %	15.30	14.77	14.00	13.15	1.23	0.261	0.591	0.899
Drip loss, %								
d1	1.80	1.75	1.71	1.67	0.21	0.705	0.858	0.964
d3	3.29	3.24	3.18	3.15	0.28	0.730	0.897	0.972
d5	4.34	4.24	4.20	4.15	0.27	0.691	0.790	0.934
d7	5.73 ^a^	5.50 ^ab^	5.34 ^ab^	5.20 ^b^	0.15	0.049	0.256	0.760

^1^ Abbreviations: (Each mean represents values from 8 replicates (18 birds /replicate)). 1,3-DAG = 1,3-diacylglycerol; WHC = water holding capacity; SEM = standard error of means; L* = lightness; a* = redness; b* = yellowness. ^a,b^ Means in the same row with different superscripts differ significantly (*p* < 0.05).

**Table 7:** Effect of 1,3-diacylglycerol supplementation in diets with different energy levels on blood profiles of broilers ^1^.

Energy	Low Energy		Normal Energy		SEM	*p* Value		
1,3-DAG	−	+	−	+				
Items						Energy	1,3-DAG	Interaction
Finish								
HDL, U/L	42.8	41.0	45.3	44.0	2.2	0.244	0.516	0.913
LDL, U/L	75.9	71.5	80.9	77.4	3.9	0.195	0.313	0.925
TC, mg/dL	135.8 ^ab^	129.3 ^b^	144.3 ^a^	139.0 ^ab^	4.0	0.043	0.171	0.879
TG, mg/dL	85.5	83.8	90.5	88.3	2.7	0.114	0.487	0.930
Albumin, g/dL	2.75	2.95	2.98	3.05	0.21	0.474	0.543	0.781
TP, g/dL	3.35	3.53	3.83	3.80	0.30	0.242	0.810	0.749
Globulin, g/dL	2.38	2.78	2.83	3.15	0.35	0.266	0.325	0.917
IL-6, pg/mL	35.48	34.48	35.25	34.13	1.54	0.856	0.505	0.968

^1^ Abbreviations: (Each mean represents values from 8 replicates (18 birds/replicate)). 1,3-DAG = 1,3-diacylglycerol; SEM = standard error of means. HDL = high-density lipoprotein; LDL = low-density lipoprotein; TC = total cholesterol; TG = triglyceride; TP = total protein; IL-6 = interleukin-6. ^a,b^ Means in the same row with different superscripts differ significantly (*p* < 0.05).

## Data Availability

The data presented in this study are contained within this paper.

## Abbreviations

1,3-DAG	1,3-diacylglycerol
FCR	Feed conversion ratio
BW	Body weight
BWG	Body weight gain
ME	Metabolizable energy
DAG	Diacylglycerol
MCT	Medium-chain triglyceride
FI	Feed intake
DM	Dry matter
N	Nitrogen
E	Energy
ATTD	Apparent total tract digestibility
WHC	Water-holding capacity
HDL	High-density lipoprotein
LDL	Low-density lipoprotein
MCFA	Medium-chain fatty acid
MDCP	Mono dicalcium phosphate
LYS	Lysine
MET	Methionine
SEM	Standard error of means
TC	Total cholesterol
TG	Triglyceride
TP	Total protein
IL-6	Interleukin-6
